# Newspaper Campaigns for Public Health

**Published:** 1914-07

**Authors:** 


NEWSPAPER CAMPAIGNS FOR PUBLIC HEALTH.—The newspapers of the country are joining the medical journals and the physicians in campaigns for better health. Almost everywhere cities, towns and villages are conducting well organized campaigns against flies and mosquitoes, and for better sanitary conditions. Dairies, meat markets, grocery and fruit stands and other places where provisions are sold come in for a liberal share of criticism when they do not come up to sanitary regulations. Speaking of the cleanliness of cities that are awake to the importance of guarding the public health, the *Beaumont Enterprise* says: "There is nothing mysterious or complicated about the modern system of sanitation that made Havana, once one of the dirtiest cities in the world, a model of healthfulness and cleanliness; that drove the deadly Chargres fever out of Panama and made the canal zone as healthful as any spot of equal size in North America; that reduced to the minimum the mortality among our troops in Vera Cruz, once the home of yellow fever and typhoid, and enabled our fleet surgeons on the Mexican station to turn in a report showing that the amount of sickness was about the same as on the North Atlantic station, say at Newport or Bar Harbor.

"Though screening against the mosquito absolutely removed the possibility of yellow fever, for only the mosquito carries that disease, while another type of mosquito carries malaria and the Chargres fever. These fevers were definitely eliminated by the elimination of the mosquito, both by screening and by the destruction of breeding places. Unquestionably, yellow fever would return if the yellow fever mosquito were allowed to breed and care was not taken to screen against them.

"Typhoid has been eliminated by screening against the fly and by abolishing his breeding places. Spread of typhoid fever is attributed almost entirely to the fly, just as spread of yellow fever, malaria and kindred fevers is attributed almost entirely to the mosquito.

"Every householder can protect his family against malaria and typhoid just as effectively as General Brook protected Havana against yellow fever and General Gorgas protected the canal zone against Chargres and malaria. It is easy for every householder to eliminate the breeding places of mosquitoes and flies and to screen effectively against the mosquitoes and flies that come from breeding places not easily reached."

